# Guest Editors’ Introduction to The Special Issue “Network Psychometrics in Action”: Methodological Innovations Inspired by Empirical Problems

**DOI:** 10.1007/s11336-022-09861-x

**Published:** 2022-04-09

**Authors:** Maarten Marsman, Mijke Rhemtulla

**Affiliations:** 1 Department of Psychology, University of Amsterdam, Amsterdam, The Netherlands; 2 Department of Psychology, University of California at Davis, Davis, California USA; 3 University of Amsterdam, Psychological Methods, Nieuwe Achtergracht 129B, PO Box 15906, 1001 NK Amsterdam, The Netherlands

In 1904, Spearman reported the positive manifold—a pattern of exclusively positive correlations among cognitive test scores—for which he proposed the *g*-factor theory of general intelligence in 1927. According to g-factor theory, general intelligence is a stable trait that cannot be directly observed but gives rise to observed test scores. The *g*-factor model quickly became the dominant theory of intelligence and, while the theory was revised and expanded, the fundamental idea went unchallenged for almost a century. Modifications to the *g*-factor theory (e.g., multi-factor models, hierarchical factor models, and their merger) were proposed and debated at length (e.g., Carroll, 1993; Gardner, 1983; Horn & Cattell, 1966; McGrew & Flanagan, 1998; Sternberg, 1985; Thurstone, 1935). Still, all these theories held intelligence and its facets to be unobservable underlying abilities. The theory’s dominance spread from intelligence to personality research, where similar debates about the underlying factor structure of personality continue to hold great interest, and to other areas of psychology where common factors came to characterize other traits, behaviors, and attitudes. Spearman’s idea now permeates many areas of psychological research.

Although alternative characterizations of the nature of psychological phenomena such as the positive manifold were proposed (e.g., the sampling model of Thomson (1916) and the gene–environment interaction model of Dickens and Flynn (2001)), the g-factor model received a challenge almost a century later, when van der Maas and colleagues published “A dynamical model of general intelligence” (van der Maas et al., 2006). This landmark paper introduced the *mutualism model*, which accounts for the positive manifold in cognitive test scores by invoking a network of mutually interacting components. The mutualism model challenged the general factor theory by suggesting that intelligent behavior—and the observed pattern of positive correlations among test scores—could evolve during development from mutualistic interactions between cognitive, behavioral, and biological factors rather than unknown common causes. That is, the very same positive manifold that was assumed to arise as a result of an underlying latent factor could, it turned out, stem from a dynamical system of positively interacting components (van der Maas, Kan, Marsman, & Stevenson, 2017; van der Maas, Savi, Hofman, Kan, & Marsman, 2019). The mutualism model of intelligence offered a radically new framework for thinking about how a psychological attribute might arise and how it might be related to its constituent parts. And, as the past 15 years have shown, the proposed network approach proved to be a promising addition to the pantheon of psychometric theories (e.g., Marsman et al., 2018).

In the 15 years since van der Maas et al.’s (2006) paper was published, its central idea has sparked an entirely new psychometric subfield. This subfield, which has come to be known as *network psychometrics, *defines psychological constructs (e.g., intelligence, mental disorders, personality traits, and attitudes) as complex systems of behavioral, cognitive, environmental, and biological factors (Borsboom, 2017; Cramer et al., 2012; Dalege et al., 2016; Savi, Marsman, van der Maas, & Maris, 2019). These biopsychosocial systems need not contain hidden units, in principle, and are defined by the local interactions between the system’s elements, which form a network. Psychometric network theory asks how psychological phenomena emerge from these local interactions, while psychometric network analysis aims to infer these local interactions from empirical data.

While the field has made great strides, it is still in its youth. The co-development of network theories and models with technical methodological advances and software releases shapes its short history. In the following sections, we briefly review this history of methodological innovations in light of the maturation of psychological network theory. We then discuss the status of the methods regarding what questions they can and cannot yet answer and how the papers in this special issue contribute to three of the most pressing open questions.

## Historical Trajectory of Network Modeling in Psychology

Borsboom (2008) took van der Maas et al.’s (2006) mutualism idea and considered it as an alternative framework for conceptualizing psychopathological disorders, suggesting that these, too, be seen as “causal networks consisting of symptoms and direct causal relations between them” (p. 1089). In the conclusion of that paper, Borsboom noted that, as of 2008, “there [was] currently no worked-out psychometric theory to go with [the network] perspective” (p. 1106), and he called for further empirical and psychometric work to elaborate on the network perspective on psychopathology. That paper marks the beginning of a concerted effort to apply network modeling theories and methods to psychological data, and to develop new theories and methods in response to those data and to the research questions of psychologists.

A few years later, seminal developments in theory and software helped the field take off. Cramer, Waldorp, van der Maas, and Borsboom (2010) took the first steps toward developing a network theory of psychopathology and showing how such a network could be modeled and visualized. Borsboom and Cramer (2013) provided examples of how to generate network visualizations from data and compute network properties like path lengths, clustering coefficients, and centrality measures. Their applied examples laid the groundwork for researchers to create network visualizations and compute network metrics from their data. At the same moment, two key software developments arrived. Epskamp, Cramer, Waldorp, Schmittmann, and Borsboom (2012) published qgraph, an R package for visualizing (and later fitting) networks in data. Shortly after that, van Borkulo et al. (2014) introduced IsingFit, an R package for fitting regularized networks to binary (e.g., symptom) data. Armed with software, applied researchers worldwide were newly able to estimate and visualize their data as networks. Many used this opportunity to spark a new way of thinking about attributes within their research areas. Early publications of this era focused on psychopathology, describing network theories of distinct disorders and using networks to explain the links between those disorders (e.g., Cramer, Borsboom, Aggen, & Kendler, (2012; Robinaugh, LeBlanc, Vuletich, & McNally, 2014; Ruzzano, Borsboom, & Geurts, 2015). Another early thread of research posited that personality, too, could be fruitfully conceived as a network of interacting components (e.g., Costantini & Perugini, 2012; Cramer et al., 2012).

While exciting, these early forays into network modeling were limited in what they were able to do: network models were, at this point, typically visualizations of correlations or partial correlations between variables observed at a single time point (although the development of longitudinal methods also began early on, e.g., Bringmann et al., 2013, Bringmann, Lemmens, Huibers, Borsboom, & Tuerlinckx, 2015). Many used $l_{1}$-regularization (“lasso”) to remove some edges to obtain a sparse network diagram. The interpretable output of these early network applications consisted of (1) a network structure, wherein edges set to zero by the regularization algorithm were interpreted as missing causal links, (2) a set of edge weights, where edges large in absolute value were interpreted as strong and potentially causal direct relations, and (3) a set of node centrality indices, which were interpreted as representing the relative importance of each variable to the system.

The development of network methodology beyond its starting point was propelled by the desire to answer specific empirical questions arising from network theory. While the initial forays into network modeling attempted to answer the most general question, “what is the structure of direct relations among variables in a multivariate dataset?,” methodologists shortly began to work on methods to answer other questions, such as “how do these structures differ across groups?” (van Borkulo et al., 2015; in press), “how does the structure of a network predict future behavior”? (Dalege et al., 2016), and “how can individual networks be used to guide clinical interventions”? (Rubel, Fisher, Husen, & Lutz, 2018; Wichers, Groot, psychosystems, ESM group, & EWS group, 2016). Several of the papers in this special issue further develop answers to these empirically motivated questions.

The early explosion of empirical network analysis applications sparked some strong criticism of the methods and the common interpretations of network models, For example, several papers questioned the stability across repeated samples of network properties such as edge weights and centrality indices (Forbes, Wright, Markon, & Krueger, 2017; Fried et al., 2018; Neal & Neal, in press). Other authors critiqued the prevalent causal interpretation of network structure and centrality indices (e.g., Bringmann et al., 2019; Hallquist, Wright, & Molenaar, 2021; Rodebaugh et al., 2018; Ryan, Bringmann, & Schuurman, 2019; Spiller et al., 2020). Yet other authors have questioned the dichotomy between networks and common factors, when in fact, both may apply to most situations (Bringmann & Eronen, 2018), and each statistical network and factor model has an equivalent in the other framework (Epskamp, Maris, Waldorp, & Borsboom, 2018; Marsman et al., 2018; Waldorp & Marsman, in press). On the estimation end, the practice of fitting network models using $l_{1}$-regularization has been shown to be suboptimal for most of the types of psychological data that network models are fit to (Williams, & Rast, 2020; Williams, Wysocki, Rhemtulla, & Rast, 2019; Wysocki & Rhemtulla, 2021), and the practice of *inferring* that edges set to zero by the regularization function are truly zero in the population (or more generally, inferring population networks are sparse when they’ve been estimated using sparse estimation procedures) has likewise been shown to be unjustified (Epskamp, Kruis, & Marsman, 2017; Williams, Briganti, Linkowski, & Mulder, 2021).

This criticism has not happened in a vacuum; methodologists have continued to develop and study new network methodologies in response to and alongside it. In response to questions about the replicability and robustness of network methodologies, researchers have begun to develop methods to quantify the uncertainty around estimated network parameters and to develop confirmatory tests for them (Rodriguez, Williams, Rast, & Mulder, 2020), as well as to investigate empirical evidence for replicability and generalizability of networks estimated on real data (Herrera-Bennett & Rhemtulla, 2021; Funkhouser, Correa, Gorka, Nelson, Phan, & Shankman, 2020). Most critics additionally offer innovative responses to their own critiques. For example, longitudinal and idiographic network methods responded to criticisms of cross-sectional network models (Bringmann et al., 2013; Epskamp, Waldorp, Mõttus, & Borsboom, 2018). New estimation methods were developed to deal with the peculiarities of psychological data (e.g., small samples, small numbers of variables, ordinal and nonnormal distributions, and dense population networks; Haslbeck & Waldorp, 2020; Williams, 2021a; Wysocki & Rhemtulla, 2021). In the face of criticism of centrality indices, researchers introduced new centrality indices and developed predictability indices (e.g., Haslbeck & Waldorp, 2018; Robinaugh, Millner, & McNally, 2016), although more development is clearly needed in this area.

When we published the call for papers for the “Network psychometrics in action” special issue in the final quarter of 2019, the field had matured a bit. We had methods that answered real empirical questions, and we had begun to grasp what our methods could or could not do. Yet, at the same time, some methodological challenges persisted. Therefore, to curate the special issue, we called for papers that “showcase how methodological innovations in the network approach that are inspired by real data can be used to answer important substantive questions.” We hoped to receive manuscripts that addressed challenges within a couple of general themes that pervade the psychological network literature. As the next section and the special issue show, we were not let down.

## Three Methodological Challenges that Impede Substantive Research | Contributions to the Special Issue

The special issue’s contributions can be organized into three research themes, focusing on unique substantive questions. The first research theme concerns the discovery of network structure: what does the population network look like, and how can we calibrate our certainty in the estimates of that structure? The second theme concerns confirmatory network methodology: how can we test hypotheses about particular edges and evaluate group differences? The third theme involves the interpretation of an estimated network: how can we identify elements of a network that are important in a conceptual, predictive, or causal sense? We consider each of these themes in turn.

## What is the Network’s Structure? and How Robust are Our Estimates of It?

The field started with a methodology that could estimate a network’s structure and parameters but could not quantify their uncertainty. As mentioned above, early research in this field revolved around network visualizations. At the time, network psychometrics was not more sophisticated than a pretty picture (Bringmann, 2016). But there is a real danger of becoming overconfident in the estimated network if one is unaware of the underlying uncertainty (Hinne, Gronau, van den Bergh, & Wagenmakers, 2020; Hoeting, Madigan, Raftery, & Volinsky, 1999). And there often is more uncertainty than researchers wish to acknowledge. For example, Mansueto, Wiers, van Weert, Schouten, and Epskamp (in press) recently showed that it is hard to recover the network structure from longitudinal data in typical sample sizes. Similarly, Fried and colleagues (Fried & Cramer, 2017; Fried et al., 2018) and Forbes and colleagues (2017; 2019a, b) initiated a discussion on the robustness of networks in cross-sectional data. With the limited data that we usually have, it is unlikely that we can be sure that the estimated network is correct and know its parameters with absolute certainty. However evident it may be, it took the field several years to develop the first methods that quantify the uncertainty of our network results. It is thus not surprising that concerns about the reproducibility of published network results have become prevalent.

The robustness of network results now firmly ranks as one of the field’s top priorities. Much of the development that addresses this research priority has focused on quantifying the uncertainty in the estimated parameters (e.g., Epskamp, Borsboom, & Fried, 2018; Jones, Williams, & McNally, 2021; Jongerling, Epskamp, & Williams, 2021). However, we believe that given the complex nature of network structure selection, more work should also focus on quantifying the uncertainty in the selected structure and address questions like, “which structures are plausible for the data at hand?” and “what impact does the uncertainty in the network’s structure have on our parameter estimates and their uncertainty?” Although some elegant Bayesian solutions have been developed that address these questions (e.g., Mohammadi, Massam, & Letac, in press; Mohammadi & Wit, 2015; Pensar, Nyman, Niiranen, & Corander, 2017; Williams 2021b; Williams & Mulder, 2020a), that have been paired with software implementations (Mohammadi & Wit, 2019; Williams & Mulder, 2020b), these solutions have received too little attention in the psychological literature. At the same time, this methodology could still use further development to be applied to the full spectrum of psychological network models and psychometric variables, and to address a broad range of empirical questions (e.g., to quantify uncertainty in centrality measures; Huth, Luigjes, Marsman, Goudriaan, & van Holst, 2021; Jongerling et al., 2021). The development of Bayesian models (e.g., prior specifications) that fit the psychological context is another area that deserves attention.

There are also concerns about the theoretical properties of uncertainty quantification (e.g., producing confidence intervals) in combination with the $l_{1}$ constraint (i.e., lasso estimation) in frequentist approaches (i.e., Bühlmann, Kalisch, & Meier, 2014, Section 3.1; Pötscher & Leeb, 2009; Williams, 2021c). Therefore, more work should focus on alternative routes to quantify parameter uncertainty, such as Bayesian or empirical Bayesian approaches or alternative forms of regularization. Some elegant Bayesian solutions have already been developed for Gaussian Graphical models (e.g., Mohammadi & Wit, 2015; Williams, 2021b; Williams & Mulder, 2020a), but non-Gaussian models have received little attention (Pensar et al., 2017, offers a recent exception).

Two papers in the special issue aim to model the uncertainty associated with estimating the network and consequently offer more robust inference. For example, Epskamp, Isvoranu, and Chueng (2022: this issue) provide a classical hierarchical approach to aggregate independent network sources into a single estimate of its topology useful for meta-analyses of Gaussian networks. The approach does not use regularization, and its standard maximum likelihood framework is blessed with familiar solutions for standard errors and confidence intervals of the estimated parameters. Marsman, Huth, Waldorp, and Ntzoufras (2022: this issue), on the other hand, offer empirical Bayes and full Bayesian solutions for selecting the structure of an Ising model, a network for binary variables, quantifying the uncertainty in the network’s estimated structure, and the associated parameters. Both contributions offer unique solutions to gauge the uncertainty in estimated networks and deliver robust network results.

## How Can We Conduct Confirmatory Tests of the Relationship Between Two Variables and Discover Differences Between Groups?

The current methodological toolbox for psychological networks is mainly exploratory. We use it to estimate a network based on the available data and then interpret the estimated network. But researchers often struggle with interpreting these networks as exploratory. For example, the absence of an edge between two variables in a lasso-estimated network is often viewed as evidence for its exclusion (Williams et al., 2021). But if the lasso edge estimate is equal to zero exactly, what evidence do we have that the edge should, in fact, be excluded from the network? The problem is that we cannot really tell. Current (frequentist) implementations of the lasso estimation procedure cannot separate *the* *evidence for absence* from* the* *absence of evidence *(i.e., that there is too little information to decide about its inclusion). Moreover, the current implementation of lasso-estimation is also not intended to be a statistical test for edge inclusion or exclusion; it is meant for selecting a single network structure but does not pit structures with a particular edge against those without that edge.

The lack of confirmatory methodology for psychological networks was a serious concern, echoed by reviews taking stock of the field (Fried & Cramer, 2017; Robinaugh, Hoekstra, Toner, & Borsboom, 2020). It is hard to formulate a cumulative science without the ability to build on what we have learned. What is the evidence for including a particular edge in the network? How do network structures compare for the data at hand? What can we say about the sign of network relations? Does the cross-sectional network hold for the population, or are there groups with a systematically different topology? In the past few years, the field has exerted considerable effort to address these questions. For example, van Bork et al. (2019; see also Kan, van der Maas, & Levine, 2019) proposed a test to identify whether data were generated from a sparse network model or a unidimensional factor model. Epskamp (2020) borrowed ideas from structural equation modeling and developed relative fit measures and likelihood-based tests for nested Gaussian Graphical models, and Williams (2021b) and Williams and Mulder (2020a) developed Bayes factor tests to assess the evidence for edge exclusion and order constraints (e.g., their sign) on the relations of these models. Van Borkulo and colleagues (in press) developed a permutation test to assess if two estimated network structures differ, and Jones, Mair, Simon, and Zeileis (2020) developed structural change tests for evaluating the impact of background variables to assess subgroup differences in the structure of Gaussian networks. For the latter, Huth et al. (in press) developed a permutation test variant suited for small sample sizes. To contrast with these classical approaches, Williams, Rast, Pericchi, and Mulder (2020) developed Bayesian solutions for assessing subgroup differences in Gaussian Graphical models (also see Williams (2021b)).

In sum, we have made great strides in developing confirmatory methods in the last three years, but the available methodology is still very limited. Where the Bayesian assessment of GGMs has received much attention, a confirmatory network methodology of non-Gaussian variables is still largely absent. Several papers in the special issue aim to fill that gap. For example, Marsman et al. (2022; this issue) offer Bayesian solutions for assessing edge inclusion for the Ising model, a network for binary variables, addressing similar questions as Williams and Mulder (2020a) did for GGMs. While Epskamp et al. (2022, this issue) offer a classical approach to gauge the heterogeneity of a GGM applied to independent datasets, Lee, Chen, DeSarbo, and Xue (2022; this issue) gauge the heterogeneity of networks of ordinal variables estimated from cross-sectional data. Lee and colleagues introduce an empirical Bayes method for estimating a finite mixture of latent GGMs to model the ordinal variables, using a new penalized Expectation–Maximization procedure to estimate the mixing weights and network parameters. Where the aforementioned approaches of, for example, van Borkulo et al. (in press) and Williams et al., (2020) assess differences between identified subgroups (i.e., observed heterogeneity), the mixture approach of Lee and colleagues allows us to assess if there are unidentified subgroups (i.e., Brusco, Steinley, Hoffman, Davis-Stober, & Wasserman, 2019). Finally, Bodner, Tuerlinckx, Bosmans, & Ceulemans (2021) recently showed how to assess the marginal dependence of two binary variables (e.g., symptom indicators) in a nonparametric way using a permutation test. In this issue, Bodner, Bringmann, Tuerlinckx, de Jonge, and Ceulemans (2022; this issue) use their method to investigate the co-occurrence of symptoms over time and construct symptom networks from the set of significant greater-than-zero values. The obtained individual network structures can then be used to reveal symptom clusters in between-subjects analyses.

## What Defining Features of A Network Foster Interpretation, Prediction, and Intervention?

Where initial network analyses focused on network plots, researchers started to wonder how to interpret their network estimates. Which relations or which nodes in the network are important? Centrality measures, borrowed from network science (e.g., Newman, 2004; Newman, Barabási, & Watts, 2006), are often used to identify the important nodes in the estimated structure. But as alluded to before, centrality measures have also received several critiques in recent years. Bringmann et al. (2019) argued that the assumptions underlying centrality measures might not apply to psychological networks. They stressed the importance of considering “for what?” when interpreting a node as important. Dablander and Hinne (2019), for example, showed that the nodes that are flagged by centrality measures might not be the nodes that are important in a causal sense. But despite these critiques and concerns, centrality measures continue to be used due to a lack of a better alternative. At the same time, centrality measures focus exclusively on the network’s nodes. But for assessing causality, it seems reasonable that not only nodes, but also particular network relations are important. In this context, Haslbeck and Waldorp (2018) proposed to use nodewise predictability—the ability of a node or group of nodes to predict others.

Four papers in this issue propose methods focused on the interpretation and use of network models. Brusco, Steinley, and Watts (2022; this issue) propose methods that work on estimated networks, cleverly reordering the rows and columns of an estimated association matrix to produce a maximally interpretable structure. These methods offer a fruitful alternative to existing centrality measures and can say something about which nodes are best able to predict other nodes, that is, which nodes are most central. In a similar vein, Golino, Christensen, Moulder, Kim, and Boker (2022, this issue) introduce a novel clustering method that we can use to identify latent topics in the time series of text data, such as Twitter data. They extend their exploratory graph analysis (Golino, & Epskamp, 2017; Golino et al., 2020) approach to discover latent topics in text data taken from interviews at a single time (Kjellström & Golino, 2019) ) to multiple time points (i.e., a time series). They apply it to estimate latent topics in Twitter data taken during the 2016 US presidential election to identify word clusters and analyze individual dynamics of separate words. Both contributions of Henry, Robinaugh, and Fried (2022, this issue) and Ryan and Hamaker (2022, this issue) offer methods that aim to characterize a network by examining its implications for intervention. Henry and colleagues bring control theory to bear on dynamic psychometric networks, showing how this method, which was developed to optimize production processes, might be used to tailor clinical interventions based on individual networks. Ryan and Hamaker, on the other hand, develop a continuous-time vector autoregressive modeling approach to constructing dynamic individual networks. This model allows the researcher to extract model-implied effects on any node in the network at any time lag. The authors show how these models can be used to form precise predictions about the impact of an intervention. These predictions form the basis for two new centrality measures, *total effect*, and *indirect effect* centrality, that indicate the importance of nodes as intervention targets.

## Software Contributions

Not only do each of these contributions push the field forward by proposing novel methods to discover and confirm network structure, summarize network properties, and use networks for prediction and intervention. All but one provides us with the software for doing so. In eight papers, four new R packages are introduced (packages: ConNEcT, Bodner et al., 2022; Bodner & Ceulemans, in press; netcontrol, Henry et al., 2022; rbinnet, Marsman et al., 2022; ctnet, Ryan, and Hamaker, 2022), two previously introduced R packages are expanded to include new methods ((psychonetrics, Epskamp, 2020; Epskamp et al., 2022; EGAnet, Golino & Epskamp, 2017; Golino et al., 2022), and one paper provides code in MATLAB and R for implementing the new method (Brusco et al. 2022).

## Closing Statement

Having offered a brief overview of the historic trends, the questions facing network psychometrics as we see them, and the contributions provided by the papers in this special issue, we leave our reader to read. We offer our heartfelt thanks to every author who contributed their fine work, and we hope you enjoy this truly excellent set of papers.
